# Therapeutic potential of nutritional aryl hydrocarbon receptor ligands for respiratory syncytial virus infection: a mini review

**DOI:** 10.3389/fmicb.2026.1887894

**Published:** 2026-08-10

**Authors:** Sydney E. Green, Antonella Casola, Aline Haas de Mello

**Affiliations:** 1 Pharmacology and Toxicology Graduate Program, The University of Texas Medical Branch, Galveston, TX, United States; 2 Department of Pediatrics, The University of Texas Medical Branch, Galveston, TX, United States; 3 Department of Microbiology and Immunology, The University of Texas Medical Branch, Galveston, TX, United States; 4 Center for Lung Disease, Inflammation and Remodeling, The University of Texas Medical Branch, Galveston, TX, United States; 5 Institute for Human Infections and Immunity, The University of Texas Medical Branch, Galveston, TX, United States

**Keywords:** aryl hydrocarbon receptor, dietary AHR ligands, gut microbiota, immune response, nutritional AHR ligands, respiratory syncytial virus, therapeutic interventions

## Abstract

Respiratory syncytial virus (RSV) infection is still a major cause of hospitalization of young children and older adults. Despite recent significant advances, critical gaps remain in prevention strategies, particularly for older infants and underserved populations, and to date there is no specific treatment available. The aryl hydrocarbon receptor (AHR) is a transcription factor initially identified as mediator of the toxic effects of environmental pollutants that was later found to be also activated by ligands derived from food, the gut microbiome, and host metabolism. AHR activation by these naturally occurring, or nutritional, ligands has been associated with beneficial physiological processes. Notably, AHR has critical functions in barrier organs such as the lungs, including regulation of immune responses to infection. AHR activation is a common strategy for many viruses to evade antiviral immunity, but in a recently published study we found that during RSV infection the AHR pathway is downregulated and plays a protective role, and the administration of the dietary AHR pro-ligand indole-3-carbinol (I3C) to RSV-infected mice led to beneficial effects. Here we review and discuss studies indicating nutritional AHR ligands, particularly compounds derived from cruciferous vegetables or generated during the metabolism of tryptophan, may offer therapeutic benefit for RSV disease, by positively modulating inflammation and reducing excessive immune responses, oxidative stress, and viral replication.

## 1 Introduction

Worldwide, respiratory syncytial virus (RSV) remains the leading cause of young children hospitalization due to lower respiratory tract infections (LRTI) (Asseri, 2025; Lopez et al., 2026). Annually, RSV causes thousands of infant deaths, with highest prevalence in low- and middle-income countries (Parums, 2025; Shaaban et al., 2025). Severe RSV infection can predispose young children to recurrent LRTI, development of asthma, and lung function impairment later in life (Zar et al., 2024). RSV is also a significant cause of severe disease, hospitalization, and mortality in older adults (Popham et al., 2025; Chaumont et al., 2026). The recent approvals of vaccines and monoclonal antibodies represent the most significant advance since the virus discovery (Ontiveros-Zuniga et al., 2026). Maternal immunization and monoclonal antibodies are now available to protect infants, and vaccines for older adults, though low uptake limits their impact on preventing severe disease (Principi et al., 2025; Murray and Chu, 2026). While critical gaps still exist in prevention strategies, there is currently no specific treatment available for RSV infection (Alvarez de Toledo et al., 2026; Lopez et al., 2026).

The aryl hydrocarbon receptor (AHR) is a ligand-dependent transcription factor involved in various physiological and pathophysiological processes, including host response to infections (Torti et al., 2021; Xu et al., 2024). AHR activation can have beneficial or detrimental consequences depending on the ligand (Haarmann-Stemmann et al., 2025). AHR was originally identified as mediator of environmental toxins effects, so its activation was perceived as detrimental. However, further investigations have determined that AHR is also activated by ligands/agonists/activators derived from food, gut microbiota, and host metabolism, and is associated with important biological processes. The discovery that AHR has critical functions at barrier organs (lung, gut, skin), including regulation of innate and adaptive immunity to prevent excessive inflammation, outlined a pathway for AHR-targeting therapeutics (Polonio et al., 2025; Coumoul et al., 2026). Tapinarof, the first AHR agonist for the topical treatment of plaque psoriasis and atopic dermatitis was recently approved, and its mechanisms of action include reduction of T helper (Th)2 and Th17 pro-inflammatory cytokines, increased antioxidant responses via the nuclear factor erythroid 2-related factor 2 (NRF2) pathway, and likely antimicrobial activity (Haarmann-Stemmann et al., 2021; Silverberg et al., 2024), highlighting the therapeutic relevance of activating the AHR pathway in certain conditions.

AHR was also found to have important roles during viral infections, but the mechanisms by which it specifically regulates immune cells and influences disease progression remain incompletely understood. Most viruses, including the severe acute respiratory syndrome coronavirus 2 (SARS-CoV-2), have been shown to induce AHR activation as a strategy to evade antiviral immunity and promote virus replication (Hu et al., 2023; Barreira-Silva et al., 2025; Grycova et al., 2025). In contrast, we recently found that during RSV infection the AHR pathway is downregulated and plays a protective role, as loss of AHR led to exacerbated inflammatory response, and the administration of the dietary AHR pro-ligand indole-3-carbinol (I3C) to mice led to beneficial effects, including anti-inflammatory and antiviral activity (Haas de Mello et al., 2025).

The beneficial effects of AHR activation have been predominantly associated with naturally occurring nutritional ligands, dietary compounds derived from cruciferous vegetables (broccoli, cabbage, cauliflower) or generated during the metabolism of tryptophan by gut microbiota and host cells (Hubbard et al., 2015; Ghiboub et al., 2020; De Juan and Segura, 2021; Huang, 2024). Considering the global burden of RSV and the lack of treatment options, we reviewed evidence indicating nutritional AHR ligands may help treat RSV infection by reducing excessive immune responses, oxidative stress, and viral replication. Our goals include raising awareness of this exciting area of investigation from our laboratory and its recent developments focused on improving outcomes for individuals affected by severe RSV disease.

## 2 RSV pathogenesis and disease severity

While RSV in most cases results in upper respiratory tract infection and mild disease, it is also associated with LRTI and severe disease including bronchiolitis and viral pneumonia. Age is a major determinant of disease severity with infants in their first RSV season and older adults being at the greatest risk for severe lower airway disease (Asseri, 2025). RSV employs mechanisms that lead to poor immune memory and susceptibility to reinfection. A short-lived immune response means within weeks or months protection is no longer achieved. However, the individual mechanisms that contribute to impaired protection are poorly characterized, in part because of the difficulty of studying local mucosal immunity in human subjects (Openshaw et al., 2017; Shi et al., 2026).

Severe RSV disease is driven not solely by viral replication but also by dysregulated host immune responses. RSV infection triggers a complex immune response involving innate and adaptive immunity and while these inflammatory mechanisms are essential for viral clearance and resolution of infection, excessive or dysregulated immune activation contributes to lung damage, airway obstruction, and severe respiratory distress, significantly influencing disease outcomes (Tripp et al., 2025; Shi et al., 2026). Dysregulated host immune responses to RSV include excessive cytokine production, Th2 and Th17 cell polarization, and impaired interferon signaling (Openshaw et al., 2017; Barnes et al., 2026). Altered regulatory T cells (Tregs), critical for limiting immune-mediated lung injury, and consequent inability to properly control the host inflammatory response have been also shown to contribute to severe RSV infection (Christiaansen et al., 2016). This dual role of inflammation, as defense against RSV invasion but contributor to disease, is important for the development of therapeutic strategies (Kombe Kombe et al., 2024; Georgakopoulou and Pitiriga, 2025).

To prevent effective host immune responses, RSV viral proteins are responsible for suppression of antiviral interferons in the host’s innate immune response which allows for immune evasion and increased infectivity (Thornhill and Verhoeven, 2020; Gutman et al., 2026). These immune-evading mechanisms from RSV can increase susceptibility to reinfection and disrupt the self-limited inflammatory response necessary for controlling virus replication and result in overactivation of inflammatory pathways leading to tissue damage and pulmonary dysfunction (Openshaw et al., 2017; Tavares et al., 2023).

Despite recent advances in prevention options, there is currently no specific treatment for RSV, and therefore an urgent need for developing therapies to treat this disease. Accumulating evidence from studies in other diseases and recent investigations from our laboratory suggest that the modulation of the AHR pathway with nutritional ligands could be explored as a novel therapeutic approach for RSV infection (Haas de Mello et al., 2025).

## 3 AHR signaling pathway

**Figure 1:**
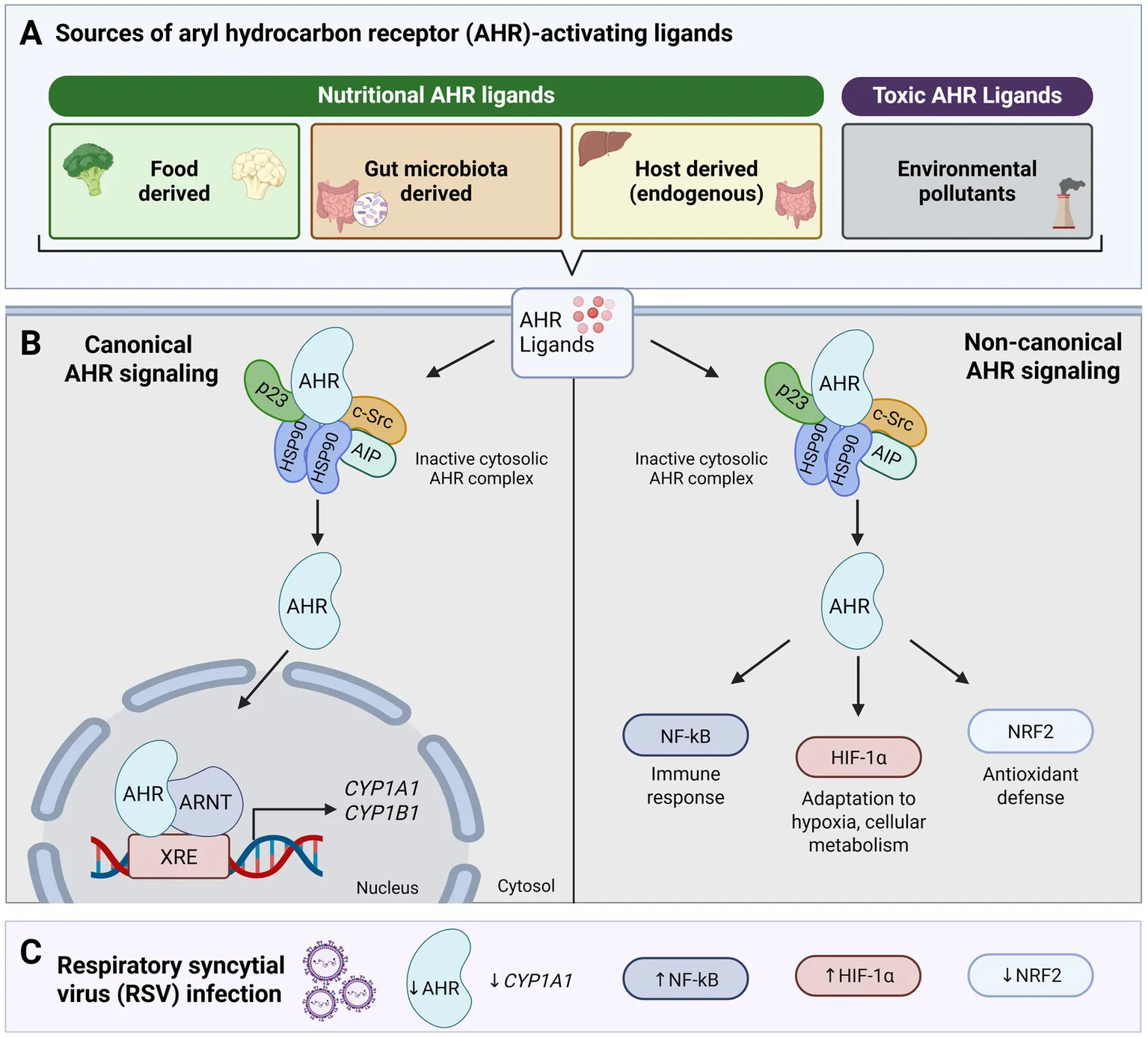
**(A)** Sources of aryl hydrocarbon receptor (AHR)-activating ligands (agonists). AHR is a transcription factor that responds to a wide variety of ligands derived from food, gut microbiome, host metabolism (endogenous), and environmental pollutants. Cruciferous vegetable-derived compounds are the main source of dietary AHR ligands, and many ligands are produced during the metabolism of the amino acid tryptophan by the gut microbiota and host cells. As the naturally occurring AHR ligands are ultimately derived from food sources, they are also known as nutritional AHR ligands. Environmental pollutants are the main source of toxic AHR ligands. **(B)** AHR signaling pathways are categorized as canonical and non-canonical. In the canonical signaling, AHR ligands bind to and release AHR from its inactive cytosolic complex to undergo nuclear translocation. In the nucleus, AHR heterodimerizes with the AHR nuclear translocator (ARNT) and the AHR-ARNT heterodimer then binds to xenobiotic-responsive elements (XREs) sites to regulate the expression of target genes, including members of the cytochrome P450 family 1 (CYP1) such as *CYP1A1* and *CYP1B1*. In the non-canonical signaling, AHR interacts with important pathways including nuclear factor kappa B (NF-κB), the central inflammation regulator, hypoxia-inducible factor 1 alpha (HIF-1α), which regulates cellular metabolism and adaptation to hypoxic conditions, and the nuclear factor erythroid 2-related factor 2 (NRF2), the master regulator of the expression of antioxidant enzymes. **(C)** Respiratory syncytial virus (RSV) was found to downregulate the canonical AHR pathway and to affect the signaling of major transcription factors involved in the non-canonical AHR pathway, including upregulating NF-κB and HIF-1α, and downregulating NRF2, all of which was shown to be important for RSV disease outcomes. HSP90, heat shock protein 90; AIP, AHR-interacting protein. Created in BioRender.

The AHR is not a cell surface receptor, but a ligand-dependent cytosolic transcription factor activated by a diverse range of exogenous and endogenous compounds. AHR-activating ligands originate from natural/nutritional sources (food/diet, gut microbiota, and host metabolism) as well as environmental pollutants including persistent organic pollutants (e.g., dioxins) and polycyclic aromatic hydrocarbons (e.g., benzo[a]pyrene) (Figure 1A). These ligands can have very different and context-dependent effects on AHR activation, leading to diverse beneficial and harmful, physiological and pathophysiological effects (Sladekova et al., 2023; Haarmann-Stemmann et al., 2025). It is noteworthy that some AHR activators have low affinity (e.g., I3C) or may not have been shown directly bind to AHR but rather function as pro-ligands or precursors that are further converted to ultimate ligands with higher binding potencies (Diao et al., 2025; Polonio et al., 2025; Coumoul et al., 2026).

AHR signaling pathways can be categorized as canonical and non-canonical (Figure 1B). When inactive, AHR is trapped in a cytosolic multiprotein complex containing two heat shock protein 90 (HSP90) molecules, AHR-interacting protein (AIP), cochaperone p23, and c-Src protein kinase. In canonical AHR signaling, upon ligand binding, AHR translocates to the nucleus and heterodimerizes with the AHR nuclear translocator (ARNT), also known as hypoxia-inducible factor 1 beta (HIF-1β), forming an AHR/ARNT complex. This AHR/ARNT heterodimer binds to xenobiotic-responsive elements (XREs) located in the target genes regulatory sequences. Among the multiple classes of AHR target genes are xenobiotic-metabolizing enzymes, including the members of the cytochrome P450 family 1 (CYP1) such as *CYP1A1* and *CYP1B1* (Bahman et al., 2024; Polonio et al., 2025). Non-canonical AHR signaling involves nuclear and cytosolic pathways, and AHR interacts with important transcription factors, including nuclear factor kappa B (NF-κB), the central inflammation regulator, hypoxia-inducible factor 1 alpha (HIF-1α) which regulates cellular metabolism and adaptation to hypoxic conditions, and NRF2, involved in antioxidant responses. Notably, depending on the ligand, cell type, and context, both canonical and non-canonical events may be involved after AHR activation (Sondermann et al., 2023).

We recently found that during RSV infection the canonical AHR signaling is downregulated (Haas de Mello et al., 2025). The presence of AHR in immune cells controls cytokine production, and the absence or downregulation of AHR can lead to excessive inflammation, ultimately causing tissue damage (Bock, 2020a, 2020b). In agreement, we found that human lung epithelial cells lacking AHR had significantly higher expression of pro-inflammatory genes and increased production of pro-inflammatory mediators following RSV infection when compared with wild-type cells (Haas de Mello et al., 2025).

RSV also affects transcription factors involved in non-canonical AHR signaling, with reported upregulation of NF-κB and HIF-1α, and downregulation of NRF2 (Figure 1C). NF-κB activation during RSV infection and subsequent upregulation of inflammatory genes play a central role in RSV-induced disease as it is initially induced to be antiviral and protective/reparative but overactivation contributes to lung immunopathology, tissue damage, and worse disease prognosis (Lotz and Peebles, 2012; Tian et al., 2018). We have shown that HIF-1α activation impacts airway function and contributes to viral replication (Morris et al., 2020; Morris et al., 2025). RSV infection is also associated with increased reactive oxygen species production and downregulation of NRF2, leading to decreased antioxidant defenses and oxidative damage of the airways, which contribute to bronchiolitis severity (Hosakote et al., 2011; Komaravelli et al., 2015; Ivanciuc et al., 2018). The canonical and non-canonical signaling showcase the functional diversity of AHR in response to certain stimuli and cellular environments.

## 4 Nutritional AHR ligands: therapeutic potential against RSV-induced disease

As RSV infection often leads to an excessive inflammatory response, which can contribute to lung injury and enhance disease severity, a therapeutic approach to limit immune-mediated tissue damage is of interest (Tavares et al., 2023; Georgakopoulou and Pitiriga, 2025). Although environmental pollutants can induce prolonged AHR activation and immunosuppression, in addition to other deleterious effects, AHR activation by nutritional or physiological ligands, i.e., natural compounds derived from food, gut microbiota, or host metabolism, predominantly have been associated with anti-inflammatory and immunomodulatory effects, supporting the concept that “you AHR what you eat” and the therapeutic applications of nutritional AHR ligands (Hooper, 2011; Lawrence and Sherr, 2012; Huang, 2024; Stockinger et al., 2024; Ran et al., 2026).

Several nutritional AHR ligands are derived from cruciferous vegetables with key examples being I3C and its derivative, 3,3-diindolylmethane (DIM). I3C and DIM are available as over-the-counter dietary supplements, with reported anti-inflammatory, antimicrobial, and antioxidant properties in various diseases (Srikanth et al., 2025). In terms of specific evidence in RSV, a recent study by our group found RSV-infected mice that were given I3C had decreased levels of pro-inflammatory cytokines, along with reduced viral replication, highlighting the anti-inflammatory and antiviral properties of I3C and its therapeutic potential against RSV (Haas de Mello et al., 2025). Evidence from other contexts that may be relevant but requires confirmation in RSV includes reports that I3C and DIM can downregulate NF-κB and HIF-1α, and reduce oxidative stress through activation of the NRF2 pathway (Srikanth et al., 2025). In addition, I3C showed antiviral activity against *in vitro* SARS-CoV-2 infection (Novelli et al., 2021; Centofanti et al., 2022) and protection against tissue damage and vascular leakage in influenza-infected mice (Major et al., 2023). DIM (combined with fish oil) improved viral pneumonia outcomes after influenza and SARS-CoV-2 infection in animal models (Kiselev et al., 2025). Importantly, I3C and DIM have been reported to regulate the balance between Tregs and Th17 cells in experimental models of colitis, type 1 diabetes, and acute respiratory distress syndrome (ARDS) (Busbee et al., 2020; Kahalehili et al., 2021; Holloman et al., 2023; Liu et al., 2023), which play a significant role in the pathogenesis of the RSV infection (Mangodt et al., 2015; Zhu and Liu, 2025).

Another main source of nutritional AHR ligands is tryptophan metabolism. Tryptophan is an essential amino acid, meaning it cannot be synthesized endogenously, and must be obtained from the diet. Higher content is found in protein-rich foods such as meat, eggs, dairy, and beans with tryptophan also being sold as dietary supplement (Hubbard et al., 2015; Ghiboub et al., 2020). Tryptophan is metabolized by the gut microbiota and host cells, and this process produces several AHR ligands, with microbiota-derived tryptophan metabolites, such as indole-3-propionic acid (IPA), indole-3-aldehyde (IAld) (also known as indole-3-carboxaldehyde), and indole-3-acetic acid (IAA) being of interest for potential therapeutic applications (Ghiboub et al., 2020; De Juan and Segura, 2021). In terms of specific evidence in RSV, IPA supplementation was investigated in a mouse model and showed to be protective, as it attenuated weight loss and reduced lung viral loads and inflammation (Antunes Fernandes et al., 2026). Similar effects were reported in IPA-treated mice infected with influenza A virus, in addition to improvements in body temperature and survival (Heumel et al., 2024; Antunes Fernandes et al., 2026). Regarding evidence from other contexts that may be relevant to RSV but requires confirmation, IAld and IAA were shown to promote IL-22 production in experimental models of candidiasis and emphysema (Zelante et al., 2013; Yan et al., 2022). Notably, IL-22 was reported to inhibit RSV replication (Das et al., 2020). IAld was also shown to alleviate inflammation by inhibiting the NF-κB signaling pathway and expression of pro-inflammatory cytokines in experimental models of osteoarthritis and colitis (Zhuang et al., 2022; Wang et al., 2023) while also demonstrating antioxidant activity in a model of intestinal inflammation (Cao et al., 2024). IAA showed anti-inflammatory and antioxidant activity in a murine macrophage cell line (Ji et al., 2020), as well as ameliorated colitis in mice, improving gut barrier function (Li et al., 2024).

Although RSV-specific evidence remains limited (Haas de Mello et al., 2025; Antunes Fernandes et al., 2026), the reported effects in other infectious diseases, including viral respiratory infections, as well as in non-infectious diseases support further investigations to explore the therapeutic potential of nutritional AHR ligands in RSV infection. In Table 1 we have provided a summary of *in vivo* preclinical studies using nutritional AHR ligands in RSV infection, in infections caused by other pathogens, and in non-infectious inflammatory diseases, highlighting the effects that could be relevant in the context of RSV infection.

**Table 1:** Summary of *in vivo* preclinical studies using nutritional aryl hydrocarbon receptor (AHR) ligands in infectious and non-infectious diseases.

Compound	Disease	Effects	References
Infectious disease: Respiratory syncytial virus (RSV)			
*Food derived (cruciferous vegetables, dietary supplements)*			
Indole-3-carbinol (I3C)	RSV infection	Attenuated weight loss, reduced pro-inflammatory mediators (BALF) and viral replication (lungs)	Haas de Mello et al. (2025)
*Gut microbiota-derived tryptophan metabolites*			
Indole-3-propionic acid (IPA)	RSV infection	Attenuated weight loss, reduced lung viral loads and inflammation	Antunes Fernandes et al. (2026)
	Influenza virus infection	Attenuated weight loss, reduced lung viral loads and inflammation, improved body temperature and survival	
Infectious disease: Other pathogens			
*Food derived (cruciferous vegetables, dietary supplements)*			
I3C	Influenza virus infection	Protection against tissue damage and vascular leakage	Major et al. (2023)
	*Clostridium difficile* infection	Ameliorated disease, increased Tregs, ILC3s, gamma delta T cells, and neutrophilic response without increasing inflammation	Julliard et al. (2017)
	Cryptosporidium infection	Antimicrobial activity (reduced parasite burden)	Maradana et al. (2023)
Diindolylmethane (DIM)*	Influenza virus infection	Prevented body weight loss and improved survival	Kiselev et al. (2025)
	SARS-CoV-2 infection	Improved disease, reduced weight loss, temperature elevation, and lung pathology	
*Gut microbiota-derived tryptophan metabolites*			
IPA	Influenza virus infection	Improved disease outcomes, reduced viral load and lowered local (lung) and systemic inflammation	Heumel et al. (2024)
Indole-3-aldehyde (IAld)	Mucosal candidiasis	Promote IL-22 production	Zelante et al. (2013)
Non-infectious disease models			
*Food derived (cruciferous vegetables, I3C/DIM dietary supplements)*			
I3C	LPS-induced ARDS	Amelioration of ARDS through regulation of Th17 and Th22 cells	Holloman et al. (2023)
		Disease attenuation and regulation of immune cell trafficking	Holloman et al. (2024)
DIM	Oxazolone-induced ulcerative colitis	Alleviated disease through Th2/Th17 suppression and Treg induction	Huang et al. (2013)
*Food derived (protein-rich foods, tryptophan dietary supplements)*			
Tryptophan	DSS-induced ulcerative colitis	Ameliorated disease, increased *Il22* mRNA and decreased pro-inflammatory cytokines	Islam et al. (2017)
*Gut microbiota-derived tryptophan metabolites*			
IAld	DSS-induced ulcerative colitis	Suppressed NF-κB inflammatory pathway	Wang et al. (2023)
	LPS-induced intestinal inflammation	Reduced pro-inflammatory cytokines and NLRP3 inflammasome activation	Cao et al. (2024)
	Cigarette smoke-induced COPD	Reduced inflammation by inhibiting the NF-κB pathway	Wang et al. (2025)
Indole-3-acetic acid (IAA)	DSS-induced ulcerative colitis	Improved intestinal barrier function and reduced inflammation	Li et al. (2024)

ARDS, acute respiratory distress syndrome; BALF, bronchoalveolar lavage fluid; COPD, chronic obstructive pulmonary disease; DSS, dextran sodium sulfate; ILC3s, group 3 innate lymphoid cells; LPS, lipopolysaccharide; SARS-CoV-2, severe acute respiratory syndrome coronavirus 2; Th, T helper; Treg, regulatory T cell; ROS, reactive oxygen species; NLRP3, NOD-like receptor family pyrin domain containing 3. *DIM formulation with fish oil.

## 5 Discussion

The RSV global burden and the lack of specific treatments underscore the need to investigate new ways to treat RSV-induced disease. Nutritional AHR ligands derived from cruciferous vegetables and produced during the metabolism of tryptophan were investigated in the context of several diseases, and have demonstrated anti-inflammatory, antioxidant, and antiviral properties, which highlight their therapeutic potential for RSV infection. Some of these compounds could restore AHR activity that is dampened during RSV infection and positively modulate RSV-induced inflammatory response and reduce oxidative stress, while ensuring viral replication is controlled.

To our knowledge, RSV-specific relevant *in vivo* evidence is currently limited to two studies, which include our own where we tested I3C (Haas de Mello et al., 2025) and a recent study that evaluated supplementation of IPA (Antunes Fernandes et al., 2026). Although the evidence for therapeutic benefit of nutritional AHR ligands in other respiratory viruses and inflammatory diseases provides reason for further investigation in RSV models, most data come from preclinical studies (cells and animal models) and is not yet validated in humans. In addition, there are still many open questions to be answered in the field, including molecular mechanisms underlying differential outcomes of AHR activation by xenobiotics vs. natural ligands, what levels and duration of AHR activation are beneficial vs. detrimental, as well as uncertain or absent data on absorption, distribution, metabolism, and excretion (ADME) for many AHR ligands (Stockinger et al., 2024; Coumoul et al., 2026).

As AHR plays diverse roles and its activation effects depend on the ligand and context, further research is essential. Ligand-based targeting of AHR is complicated and besides canonical signaling, AHR exerts diverse effects through non-canonical pathways as it interacts with important transcription factors that regulate immune responses, adaptation to hypoxia, and oxidative stress (Sondermann et al., 2023), which are important in the context of RSV and affect infection outcomes (Komaravelli et al., 2015; Morris et al., 2025). Inflammation is a necessary part of the immune system’s response to infection, and although reducing excessive immune responses might confer benefit to severe disease patients, immunosuppression must be considered, as reduction in lymphocyte recruitment and activation can delay viral clearance (Tavares et al., 2023). Although AHR activation by natural or nutritional compounds have been shown to modulate beneficial immunological responses and to have immunoregulatory effects instead of the profound immunosuppression or immunotoxicity associated with environmental pollutants (Esser et al., 2018; De Juan and Segura, 2021), the effects on RSV clearance should always be evaluated. An ideal therapy for RSV would promote balance between effective viral clearance and minimizing immune-mediated lung damage (Georgakopoulou and Pitiriga, 2025).

Moving forward, additional preclinical studies in RSV models are needed before clinical studies become a realistic option. Repurposing dietary supplements seem to have major advantages since pharmacological and safety profiles may have already been demonstrated for other indications (Coumoul et al., 2026). I3C and DIM have been tested in clinical trials for cancer, making them promising candidates for repurposing (Reyes-Hernandez et al., 2023; Srikanth et al., 2025). Prophylactic dietary interventions, such as increased consumption of cruciferous vegetables, could potentially also be explored. To allow future translation from model systems to humans, it is imperative to use experimental models that take into consideration the critical physiological functions of AHR and the distribution of the agonist in different tissues, as agonists of similar potency have different ADME properties (Stockinger et al., 2024).

In conclusion, future investigations into the mechanisms and physiological implications of agonistic nutritional AHR ligands could pave the way for discovery of an urgently needed therapeutic option against RSV and improve outcomes for the affected vulnerable populations. However, as AHR activation cannot be generalized as beneficial or harmful and is highly ligand-dependent, using nutritional AHR ligands as treatment for RSV will require more research.
